# Risky Business: The Function of Play in a Venomous Mammal—The Javan Slow Loris (*Nycticebus javanicus*)

**DOI:** 10.3390/toxins13050318

**Published:** 2021-04-28

**Authors:** Meg Barrett, Marco Campera, Thais Q. Morcatty, Ariana V. Weldon, Katherine Hedger, Keely Q. Maynard, Muhammad Ali Imron, K. A. I. Nekaris

**Affiliations:** 1 Nocturnal Primate Research Group, School of Social Sciences, Oxford Brookes University, Oxford OX3 0BP, UK; megbarrett29@gmail.com (M.B.); mcampera@brookes.ac.uk (M.C.); thais.queiroz.morcatty-2018@brookes.ac.uk (T.Q.M.); a.weldon@brookes.ac.uk (A.V.W.); research@littlefireface.org (K.H.); keely.maynard@hotmail.com (K.Q.M.); 2 Department of Forest Resources Conservation, Universitas Gadjah Mada, Yogyakarta 55281, Indonesia; maimron@ugm.ac.id

**Keywords:** social learning, positional behavior, development, animal weaponry, immature, function, intraspecific competition

## Abstract

Immature mammals require opportunities to develop skills that will affect their competitive abilities and reproductive success as adults. One way these benefits may be achieved is through play behavior. While skills in developing use of tusks, antlers, and other weapons mammals have been linked to play, play in venomous animals has rarely been studied. Javan slow lorises (*Nycticebus javanicus*) use venom to aid in intraspecific competition, yet whether individuals use any behavioral mechanisms to develop the ability to use venom remains unclear. From April 2012 to December 2020, we recorded 663 play events and studied the factors influencing the frequency of play and the postures used during play in wild Javan slow lorises. Regardless of the presence of siblings, two thirds of play partners of young slow lorises were older and more experienced adults. Young lorises engaged in riskier behaviors during play, including using more strenuous postures and playing more in riskier conditions with increased rain and moonlight. We found that play patterns in immature lorises bear resemblance to venom postures used by adults. We suggest that play functions to train immature lorises to deal with future unexpected events, such as random attacks, as seen in other mammalian taxa with weapons. Given the importance of venom use for highly territorial slow lorises throughout their adult lives and the similarities between venom and play postures, we cannot rule out the possibility that play also prepares animals for future venomous fights. We provide here a baseline for the further exploration of the development of this unique behavior in one of the few venomous mammals.

## 1. Introduction

Different types of weapons, such as antlers, tusks, horns, canines, and toxins have evolved in numerous animal taxa [1,2] In many of these species, weapons are used to defend vital resources that directly or indirectly translate into mating opportunities [2]. The proposed reasons for the evolution of such characteristics include intrasexual selection, defense against predators, and competition over sleeping sites and food [3]. Typically, in species that possess such weapons, sexually dimorphic male and female adults also assume distinctly different roles. The evaluation of adult behavior requires a clear understanding of ontogeny [4,5,6]. The causation and function of species-specific behavior patterns are better understood through the comparative study of behavioral development [5]. One way to study the development of adult behaviors is through play, as it is hypothesized that play may be one way in which young individuals learn and practice behaviors that will be useful as adults [7,8].

Play among weaponized mammals typically reflects adult behavior and has the potential to be risky. For example, play patterns in Siberian ibex (*Capra ibex sibirica*), a species in which males possess larger horns than females, resemble those observed during adult male aggressive interactions. Young males engage in more play than females, suggesting that play promotes the development of adult behaviors that are linked to reproductive success [7]. In the American porcupine (*Erethizon dorsatum*), defensive play movements duplicate what transpires when an individual actively defends itself. These include attempted protection of the head, complete erection of quills and hair, and/or attempts to crowd the quills against another individual. Play in these species can be severe enough to lead to death [9]. Further, play among elephants includes mounting, chasing, pushing, trunk wrestling, mock charging and vigorously sparring head-to-head. Play sparring in young male elephants has the ability to escalate into more aggressive bouts using tusks and charges that resemble serious male-male fights that can result in serious injury [10]. Therefore, in animals that possess dangerous adaptations, play may be important to learn how to mitigate use of strength, weapons, and toxins.

Toxins including venom have also evolved as a type of weapon [11]. Only a handful of venomous mammals have been identified, and these include *Blarina* spp., *Neomys* spp. and *Solenodon* spp. (Eulipotyphla); vampire bats *Desmodus rotundus* (Chiroptera); platypus *Ornithorhynchus anatinus* (Monotremata); and slow lorises *Nycticebus* spp. (Primates), with venom used as a weapon by the latter two taxa [12]. Play is rarely mentioned in studies of insectivores and is thought to be uncommon; evidence for play in the platypus is weak and strictly anecdotal [13]. A single captive study on the behavioral development of seven immature vampire bats revealed that social play includes behaviors such as mounting, wrestling, and chasing. It is thought that these behaviors not only function to aid in complex social interactions but also in the development of aggressive behaviors as adults [14]. Beyond this example, little is known regarding play in venomous mammals, and thus, requires further exploration.

There is no consensus on the exact function of play, though researchers agree that it is both adaptive and functional, supported by evidence that play is fundamental to the development of normal social behaviors [5,6,15,16] and motor skills [5,6,17,18,19]. Most venomous taxa (e.g., invertebrates, reptiles) tend to give birth to multiple offspring with limited parental care and therefore do not have a long period of dependency to learn survival skills. For venomous animals with altricial offspring, it may be necessary to learn how to use venom and play may be a way to gain this experience early in life. Multiple theories regarding the function of play have been proposed suggesting that play serves numerous overlapping functions that aid in individual development [6,15]. An early and predominant theory of play that can be linked to learning to use venom is the “motor training hypothesis”, which states that play exists to facilitate the development of species-typical behaviors by refining the motor skills required in order to perform such behaviors [20]. If this is valid, then it can be expected that play patterns in young animals will resemble behavior patterns in adults, such as those used during aggressive or sexual interactions. This pattern has been shown in numerous mammal species that possess weapons such as fangs or horns, including olive baboons (*Papio anubis* [17]), Siberian ibex [7], Scimitar-horned oryx (*Oryx dammah* [21]), Cuvier’s gazelle (*Gazella cuvieri* [22]), and bighorn sheep (*Ovis canadensis* [23]).

Play tends to occur most frequently in immature individuals [6,20,24] and, therefore, coincides with the development of the cerebellum, a part of the brain that is important for motor performance as well as the motor neurons of the peripheral nervous system, providing further support for the motor-training hypothesis [25]. Typically, among mammals, including those possessing weapons, immature individuals are recorded as play fighting with others of a similar age (e.g., chacma baboons, *Papio cynocephalus* ursinus [17]; bighorn sheep [23], sable antelope, *Hippotragus niger* [26]; chimpanzees, *Pan troglodytes* [18]). Play fighting often involves competing over the same areas of the body that are bitten or struck during serious fighting, in a reciprocal manner between the players [27]. Preference for play fighting with individuals that are at a similar stage of development may provide opportunities to make social comparisons and test out the relative abilities of others. Further, if play fighting is used to practice and refine combative strategies, then the preference for play partners that are closest in age seems to reflect a preference for those that are similar in skill and strength. As play appears to be necessary for development, natural selection should favor individuals who select play partners that aid in the optimal development of the individual [26]. Therefore, examination of play partners may provide information regarding the adaptive significance of play fighting in regard to weapon use.

The second leading hypothesis regarding the function of play that may link to venom use is the “training-for-the-unexpected” hypothesis. This hypothesis states that play helps to train animals to overcome stressful or unexpected situations by allowing them to develop flexible emotional and kinesthetic responses to events that involve a loss of control [28]. These benefits are achieved because animals expose themselves to a variety of actions, such as being knocked over or falling during play, which leads to unpredicted outcomes [28]. This type of play may also help individuals learn to respond to more serious attacks from others, including continued fighting through painful bites or toxic sprays. During play, individuals may create or seek ways in which they lose control or are at a disadvantage [28,29,30]. This may be done by playing with more experienced and stronger play partners (i.e., older individuals) or by moving in less stable or exaggerated ways that might compromise their postural stability [28,29,30,31]. For example, Siberian ibex kids prefer to engage in play on sloped terrain rather than on flat terrain, even though the former results in greater risk [31]. 

**Figure 1:**
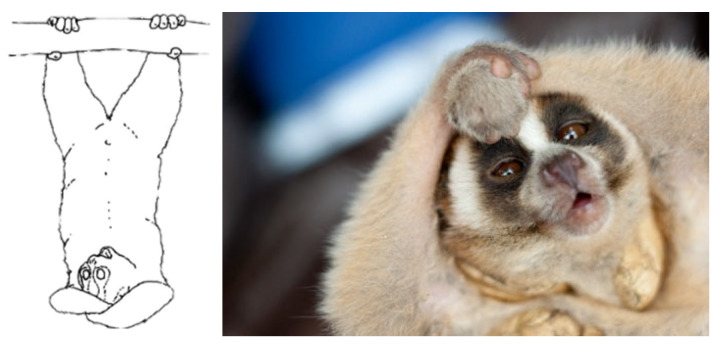
Drawing of a slow loris engaged in the venom pose and photo of a loris held by a researcher with the arm positions of the venom pose; the arms are held tightly clasped above the head allowing the animal to mix saliva with oil from their upper arm brachial gland. (Courtesy of Little Fireface Project.)

As the only venomous primate, which also shows regular social interactions [32], slow lorises provide an excellent opportunity to study the function of play and how it might relate to developing skills to deliver venom during real fights. When a slow loris is threatened it produces a compound venom in its saliva and brachial gland. In the wild, to inject venom, a slow loris combines these fluids in the mouth by raising its hands over its head while hanging bipedally (known as a venom pose; see Figure 1), combining saliva with brachial gland exudate [12,33]. This position, with the hindlegs securely gripping a substrate, allows the animal to grapple another and bite with the toothcomb, comprised of powerful procumbent anterior teeth, thus injecting the venom into an aggressor [33,34]. Slow loris bites containing venom have been shown to be dangerous, resulting in severe, necrotic wounds [11,12,33]. They not only result in serious wounding or death of wild conspecifics but are one of the most common causes of death in captive lorises. Slow loris venom can not only kill a variety of small-bodied animals but can also cause anaphylactic shock and even death in humans [34]. A long-term wild study on the function of slow loris venom revealed that 20.4% of all lorises captured exhibited fresh bite wounds, including necrotic wounds to the head as well as loss of ears and digits [11]. Many aggressive interactions lead to the use of venom in slow lorises [11,32]. During such aggressive interactions, individuals also, before adopting suspensory venom poses, engaged in chasing, wrestling, and biting. Venom use has also been shown in immature lorises, with younger individuals being more aggressive, as expressed also by a more contrasting aposematic coloration [32]. The toothcomb that injects the venom is fully developed within 220 days. In a reported case of anaphylactic shock in a human, the patient had been bitten by a juvenile slow loris [34]. 

While venom in slow lorises has been shown to be a weapon used in intraspecific competition, males and females differ in the resources they defend [11]. Males use venom to defend reproductive females for access to mating opportunities, whereas females use venom to defend critical resources, such as food trees and sleeping sites, as well as defending offspring. Males exhibit a significantly higher presence of wounds than females. Slow lorises display a promiscuous mating system and lack a breeding season. This allows males more opportunities for contests and, therefore, results in higher rates of wounding [11,32].

Javan slow lorises (*Nycticebus javanicus*) are nocturnal, medium-bodied (~905 g) primates that lack sexual dimorphism and live in small, family groups consisting of an adult male, an adult female, and one to four offspring. Young offspring are cared for by all members of a family group and both males and females disperse at anywhere from 18 months to just over three years [35]. From 12 months onward, slow lorises may investigate the edges of their home ranges, and as a highly territorial species, must already have the ability to fight strangers [11,32]. Play behavior has rarely been recorded in wild nocturnal primates; in three species sharing the same social organization as Javan slow lorises, limited observations of play involved only juvenile individuals and the mother (*Aotus azarai azarai* [36]; *Loris lydekkerianus* [37]; *Tarsius syrichta* [38]). Play behavior in slow lorises has been recorded in captivity across all age classes [39,40], but until now, wild play has not been described in detail. Further, much of the data supporting theories of play come from non-venomous, diurnal, sexually dimorphic species living in large groups. To gain a more holistic view of play and its evolutionary significance, it is critical to study play behavior in a broader range of species, specifically those that deviate from the formerly mentioned social structures [41]. 

The aim of our study is to provide the first detailed description of play in a wild, venomous mammal and to examine if play potentially provides an avenue to improve venom-use skills in the framework of two hypotheses regarding play. We thus examine the age of play partners. If the motor-training hypothesis is supported, we predicted that immature slow lorises would engage in play mostly with siblings, as they are closest in age. If the training-for-the-unexpected hypothesis is supported, we predicted that immature animals would choose older, stronger and more experienced play partners. As this hypothesis also suggests that animals must deal with unexpected stressful events, we also predicted that animals will self-handicap by playing more often at riskier times, when the moon is bright or while it is raining when substrates are less safe. Several nocturnal mammals, in fact, tend to avoid bright nights as the predation risk increases (although they can also have benefits such as increased foraging efficiency) [42]. Slow lorises tend to be less active during bright nights, contrary to other primates [43]. For both hypotheses, we expected play to resemble behaviors used during venom use and intraspecific conflicts, including in the use of the suspensory posture that characterizes venom use. We thus predicted that immature individuals would engage in increased suspensory postures during play relative to non-suspensory postures, which place them at more risk from falling than non-suspensory postures, and potentially help them to refine combative strategies.

## 2. Results

### 2.1. Play Partners

We collected a total of 663 play events; in 655 of these we could identify partners. Play partners’ ages were as young as 67 days and as old as 4535 days. The social network analyses revealed that all combinations of play partners occurred. Play almost always took place in pairs, with an immature paired with the adult male (n = 224), the mother (n = 185), or a single sibling (n = 185). Adults played on their own only 23 times. Three or four individuals playing together was observed only 38 times, comprising an immature individual with the adult male and female (n = 25); with one parent and a sibling (n = 12); and with two other siblings (n = 1).

### 2.2. Play Patterns

Play sessions averaged 27 minutes, ranging from just under 5 minutes to over 155 minutes. In 136 play sessions, we collected detailed contextual information of the patterns of play. After play was initiated through two animals approaching each other, play partners engaged in combinations of the following behaviors. Animals typically hung upside-down by two feet and engaged in wrestling and grappling (n = 51), intertwining themselves (n = 12), and focusing attacks and bites on their opponent’s head/neck (n = 19) or feet (n = 7). Using their arms, they attempted to grab at the other individual (n = 8). One individual sometimes hung off of their opponent or clung to them rather than hanging off a branch (n = 9). One individual may try to climb on top of their play partner (n = 11). Lorises also engaged in chase play in which one individual quickly followed another (n = 13). Animals occasionally emitted audible play grunts (n = 10). We also observed falling while playing (n = 3). Of 447 capture records of slow lorises throughout the study period, we captured infants and juveniles only 55 times (20 females and 16 males), and only four of these showed wounds. All of these were males (25% of the males had wounds) and all the wounds were to the head. 

### 2.3. Postures during Play

**Figure 2:**
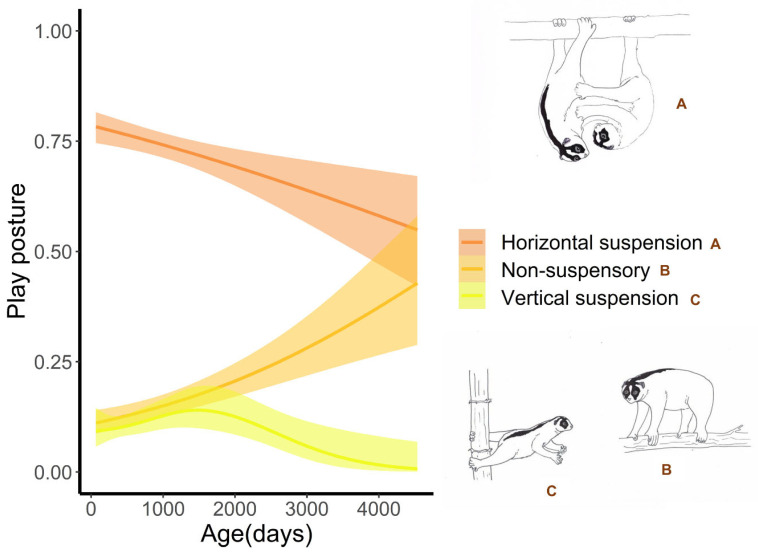
Relative use (proportions) of play postures in relation to age in Javan slow lorises (*Nycticebus javanicus*) from April 2012–December 2020 in West Java, Indonesia. Lines indicate fit functions based on Generalized Additive Mixed Models; shaded areas indicate 95% confidence intervals.

Lorises mainly played using horizontal suspension postures (74.0%), followed by non-suspensory postures (15.7%), and vertical suspension (10.3%). The horizontal suspension postures were more frequent in young individuals and gradually decreased with age, and the proportion of non-suspensory postures was less frequent in young individuals and gradually increased with age (Figure 2A,B). The proportion of vertical suspension postures did not follow a significant pattern with age (Table 1; Figure 2C). Play postures were also influenced by the play partner, with siblings playing more often in non-suspensory postures and less-often in horizontal suspension postures than in other relationships (i.e., partner or parent-offspring) (Table 1). Siblings also played more often in vertical branch suspension and less often in horizontal suspension postures than when mother-offspring played. The play postures when siblings were playing were not significantly different than the play postures when adult male-offspring were playing. 

### 2.4. Rain and Moonlight during Play

**Figure 3:**
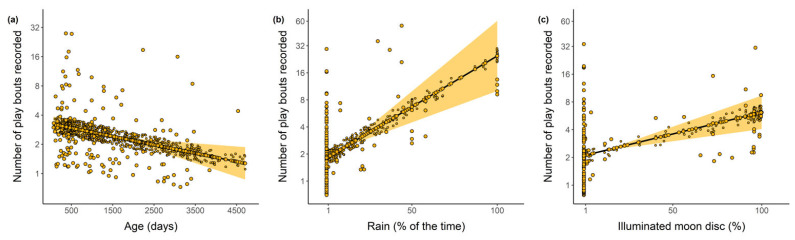
Relationship between the frequency of play bouts recorded for Javan slow loris (*Nycticebus javanicus*) and age of focal individuals (**a**), the occurrence of rain (**b**) and the intensity of moonlight (**c**).

Both rain and moonlight positively influenced the frequency of play, in that during rainy periods and with high illumination by the moon, the focal individual played more often. Conversely, age negatively influenced the frequency of play, in that the older the individual the less it played. Group size and sex did not have any influence and were not retained in the best model (Figure 3; Table 2). 

## 3. Discussion

Our study is the first to describe detailed play in a wild, venomous mammal and to investigate its functions. We found varying support for both the motor training and the training-for-the-unexpected hypotheses. For the motor training hypothesis, we found that play closely resembled aggressive interactions including the postures and patterns used to inject venom. For the training-for-the-unexpected hypothesis, not only did slow lorises more often play with adults than same-aged siblings, but they also played during riskier periods when the moon was brighter and during rainier nights when substrates are more slippery. Although we cannot say definitively that play in slow lorises functions as practice for future venom use in agonistic interactions, aspects of play meet the definition of play fighting. Namely, although animals grapple in the same postures as fighting adults, it does not look serious and does not lead to injurious or lethal bites [27]. Furthermore, animals can swap vulnerable positions and engage in risky behaviors, both of which have been linked to fighting practice in other weaponized animals [27,44].

### 3.1. Play Partners

Consistent with age trends in play for other mammals [6,20,24], immature individuals played most often. Although they played with all age classes, they engaged in play with adults for 62% of observations, with one third of all observations comprising play with adult males (e.g., social fathers). In slow lorises, males are the most aggressive and most subject to venomous bites [11]. They thus could be the ideal partners to learn the rules of play and, subsequently, aggressive conflicts involving venom use. After adult males, young slow lorises played the most with one or more other immature individuals. Among mammals, individuals typically show a preference for similar-aged and/or similar-sized play partners [45]. For example, similar-aged play partners were preferred in young sable antelopes, another weaponized mammal in which age correlates with body size and body size is roughly proportional to strength, suggesting a preference for partners that are similar in skill and strength [28]. These findings suggest that play is used to practice and improve combative strategies. Therefore, in support of the motor-training hypothesis, immature slow lorises may engage in play most often with their similarly skilled siblings to improve and refine their own combative abilities that will be useful in agonistic adult contexts [28], such as learning to meter and use venom, whilst refining these skills with their larger parents, supporting the training-for-the-unexpected hypothesis.

### 3.2. Play Patterns

Play bouts regularly lasted more than 20 minutes, with the range of bouts (5 min to 2 h and 35 min) which is similar to those of fights (3 min to 3 hours and 55 min) [11]. During play, lorises mainly hung bipedally in the same posture as the venom pose while engaging in rough-and-tumble play (i.e., grappling/wrestling). Targets of play bites were most often the head or neck area, which are also the most frequent targets of bites involving venom use during aggressive competition [11,32]. Previous studies have shown that venomous bites to the head area are more likely to heal, leaving only a scar, whereas other areas such as ears and digits are more likely to be lost following venomous bites [32]. Therefore, play bites to the head may be less risky in avoiding serious injury whilst still allowing young individuals to learn how to use their venom during aggression. Indeed, we only recorded four minor injuries to young lorises, all of which were to the head. As young lorises are parked towards the center of their home range in dense thickets, and intruders are rare, it is more likely these bites result from play, especially since biting was common during play. These wounding patterns were distinctly different from the regular more severe wounds seen in older animals, comprising 33% of females and 47% of males [11]. We also observed individuals to chase each other during play bouts. These play patterns resembled those observed in adult slow lorises engaging in agonistic interactions in the lead up to the use of venom, or to escape being bitten. It is thought that rough-and-tumble play aids in the development of skills used in aggressive interactions as it is typically made up of elements similar to those observed in adult agonistic interactions. Further, fighting skills improve with experience and practice, and therefore, many researchers agree that this type of play functions to improve an individual’s ability to successfully compete as adults [17,28].

Play patterns in other species that possess weapons have also been found to resemble aggression. For example, during aggressive interactions, American black bears (*Ursus americanus*), which are equipped with a dual weapon system (teeth and claws), may vigorously swipe their claws at their opponent’s body, which can result in serious injuries. While play-fighting, bears use a form of inhibited clawing, as the claws are not flexed down, and the opponent is hit with the pad of the foot rather than the claws [46]. Young paper wasps (*Polistes dominulus*) engage in play fighting that resembles dominance interactions that they will use later in life [44]. It is thought that this behavior allows wasps to assess their dominance potential without any negative outcome. Wasps that showed higher rates of play behavior seemed to enhance their physiological status, thus increasing their probability of becoming alpha [44]. Therefore, the intense behaviors observed in slow loris play, such as biting or chasing, are likely used to create or sustain a competitive edge during play bouts that allow young individuals to practice for future aggressive interactions involving venom use that require the defense or acquisition of resources.

### 3.3. Postural Behaviour during Play

Slow lorises mainly played in horizontal suspensory postures where most strength is required in the hindlimbs. Young lorises also engaged in higher rates of vertical suspension, compared to older individuals, where the center of gravity has to be held in a perpendicular line to a substrate requiring much more core strength [47]. That posture decreased as individuals aged, specifically around the time of dispersal when individuals are making larger forays from their natal range [35]. Among primates, younger individuals typically have a more diverse positional repertoire [48]. Suspensory behaviors, however, might be linked to a lower body weight as in young juvenile capuchin (*Cebus* spp.) monkeys that increase suspensory behaviors involving their prehensile tail during social behaviors [49]. It has been suggested that results such as these highlight the importance of perfecting riskier and acrobatic positional modes during development, as such postures are typically linked to those that are beneficial in accessing food resources and/or escaping or avoiding predators [49]. Slow lorises are considered arboreal quadrupeds and do not have the ability to leap [47]. Further, in a study of slow loris aggression, such interactions often result in one or more individuals falling from a tree [11]. It seems likely then that play in young slow lorises may aid in training them to be better equipped to deal with an arboreal lifestyle, as arboreal animals must cope with unstable and unpredictable substrates [50]. Therefore, in support of the training-for-the-unexpected hypothesis, play may function in allowing individuals to learn how to respond to unexpected falls and to adapt to the demands imposed by unstable arboreal substrates by promoting the development of dynamic and static flexibility. Older animals may therefore engage in play more often in non-suspensory postures because they are more experienced in coping with unexpected events and unstable arboreal strata.

### 3.4. Risky Behaviours during Play

When considering overall age of play partners, we found that immature slow lorises played more often with their parents than with other siblings. We note here that not all young animals were collared, and thus the proportion of play bouts between immatures is likely under-represented. Still, this large amount of play bouts between youngsters and older play partners suggests that play may also function in support of the training-for-the-unexpected hypothesis. In this sense, slow lorises may put themselves at a disadvantage by choosing partners that are older, larger and more experienced. The younger individual may therefore benefit from the experience of coping with being at a disadvantage during the play bout, as older individuals may play more roughly [29,30]. In the Siberian ibex, a species in which adults possess large horns, kids were also found to choose play partners that were slightly larger, suggesting that they choose partners that would offer the most vigorous play [7]. Thus, younger lorises seem to choose more experienced play partners that will offer a more challenging play bout and allow them to train to deal with future risky events by gaining or improving their own skills from older, more experienced play partners. As venomous bites are seen so often in dispersing and older individuals during conflict, and have the potential to be deadly [11,33], young lorises should benefit by learning to mitigate and counteract such risky attacks, and play seems to be a principal way in which to do this.

Rough-and-tumble play, the main way in which slow lorises play, is considered to be a risky behavior, as individuals may fall, become injured, or experience a loss of control [27]. We found that slow lorises played in disadvantageous ways, in that individuals played more often when it was raining. It may be riskier to play during rainfall, as an individual’s vision may be compromised, and wet and slippery branches can impact an animals’ speed or ability to grasp the surface [51]. Thus, playing during rain likely allows young slow lorises to learn to deal with less-than-optimal environmental conditions. Although we did not include humidity in this study, previous work detected an increase in loris activity when the humidity is high, probably as a response to a higher availability of insects, part of loris’s diet [43]. Therefore, it is advantageous for the juveniles to be trained for those foraging opportunities. The same principle applies to an increase in play with increasing moonlight. Given that nights with higher intensity of moonlight may make lorises more easily detected by predators, immature individuals need to learn how to refine their movements while avoiding predation in such conditions [42]. Likely as an anti-predatory behavior, loris activity was reported to overall decrease with an increase in moon luminosity [43]. However, an exception to that pattern was found for pygmy loris (*Nycticebus pygmaeus*) on notably warmer nights, when activity can still be high in bright moonlight [43,52]. Consequently, prior training to deal with susceptibility to predation when activity is needed on warmer, bright nights may be advantageous for the juveniles, and therefore, plays offer this training opportunity. In addition, the reduction in the displacement expected for slow lorises in brighter moonlight may bring family groups closer to the core of the range, further facilitating social interactions [43].

We also observed that individuals would occasionally hang off of their play partner rather than hang onto a substrate and would sometimes swing or twist their bodies during play. In this view, play results in an increased range of movements that can be used to recover from sudden shocks such as falling, being knocked over, or pinned down while also enhancing the abilities of an individual to cope emotionally with such unexpected situations, such as facing dangerous stimuli or future venomous attacks [29]. 

## 4. Conclusions

For animals that possess weapons, play has been suggested to comprise an important role in learning to use those weapons both physically and cognitively [20,23]. Play behavior is far less documented in animals that use toxins as weapons, but where research has been done, play can play an important role in refining toxin use in social contexts [44]. Here, we provide novel data on slow loris play, showing its potential role in motor development and in social relations. In particular, we show the similarities of play with agonistic and venom use behaviors, the unusually large amount of play with more aggressive and experienced adult males, and the refining of play during risky periods. Through this study, we show many features of play behavior to resemble venom use in slow lorises. Postures used in both play and venom use were similar, in that individuals hung bipedally upside down off a horizontal substrate. Rough-and-tumble play fighting often mimics aggressive interactions through biting, hitting, and chasing [27], and this was observed throughout this study. Targets of bites were the head and neck area, which are two of the same targets used in aggressive interactions involving venom use. These observations all suggest that play fighting may help lorises train for future agonistic encounters.

Given the importance of venom use for highly territorial slow lorises throughout their adult lives, we feel we have provided a baseline for further exploration of the development of this unique behavior in one of the few venomous mammals. Gaining further support for the role of play fighting in venom use for a secretive nocturnal mammal will be challenging, but with these data as a baseline, we suggest examining aggressive interactions involving the use of venom in adults. Calculating how many times each adult wins a fight and comparing it to the amount of time the individual engaged in play when younger could provide key support to this hypothesis.

## 5. Materials and Methods 

### 5.1. Ethics Statement

We collected all data in compliance to guidelines and ethical practices provided by the Association of the Study of Animal Behaviour and the Indonesian Ministry of Science and Technology, RISTEK (802/FRP/E5/Dit.KI/VII/2018). Animal capture and radio collaring followed a protocol approved by the Animal Ethics Subcommittee at Oxford Brookes University and followed the American Society of Primatologists Principles for the Ethical Treatment of Non-human Primates.

### 5.2. Study Site

We collected data at the Little Fireface Project (LFP) field site, a long-term project where a wild population of Javan slow loris has been studied continuously since 2012. The area is an agroforest habitat located on Mount Putang near the village of Cipaganti (S 7°6′6″ to 7°7′ and E 107°46′ to 107°46′5″), West Java, Indonesia [43,47].

### 5.3. Observation Methods

We conducted focal observations on Javan slow lorises six nights a week, from 18:00–0:00 and/or from 0:00 until the focal individual entered a sleep site. We caught animals by hand and did a series of systematic measurements, including recording the presence of fresh wounds [11]. We followed 56 individuals via an antenna (Yagi, Biotrack, UK) and receiver (Sika, Biotrack, UK) from April 2012–December 2020 (Appendix A). We conducted behavioral observations using a headtorch equipped with a red filter, as red light has the least impact on nocturnal animals’ behavior [43]. We recorded behavioral and social data using a detailed ethogram [47,53], using instantaneous focal sampling at 5-min intervals. We considered all occurrences of play behavior and we included ad libitum detailed descriptions where possible [54]. We analyzed observational play data collected from April 2012–December 2020 in lorises of all age classes. 

### 5.4. Play Observations

We defined play behaviors as mock fighting (rough-and-tumble) with low intensity biting, hitting, and/or clasping without agonistic vocalizations or intent [53]. When a focal individual was observed to engage in play, we recorded the individual(s) that the focal played with if they could be identified and the posture the individual was in. Postures included “horizontal suspension”, “vertical suspension”, and non-suspensory (i.e., standing, sitting, or walking). Horizontal suspension occurs when an animal’s weight is supported by the strength of the legs and possibly one arm, whereas vertical suspension is a more rigorous posture whereby the animal’s weight is perpendicular to a substrate supported by its core strength, with only two legs holding on to the substrate [47]. We recorded any details and patterns observed during play bouts in ad libitum notes, when possible.

### 5.5. Data Analysis

To determine the relationship between play partner selection, we conducted, e.g., social-network analysis using Gephi v. 0.92 [55]. We only included play events (n = 655) where we could identify all individuals. As we did not always know which individual initiated play, we used undirected nodes with each age category as a separate node. We tallied the number of instantaneous sample points of play for each play partner pairing (i.e., sibling, mother, or adult male), which we used as weighted edges [56]. We use the term “adult male” here rather than "father" as we were not able to genetically assess parentage.

We determined whether the posture of play changed with age via Generalized Additive Mixed Models (GAMMs) with posture categories (horizontal suspension, vertical suspension, non-suspensory) as dependent variables fitted to binary distributions, and age and relationship between play partners (siblings, adult male‒offspring, mother‒offspring, other, i.e., play between pair mates or between offspring and both parents) as the independent variables, including individuals as random effects. We tested the posture of play separately by setting the selected posture as 1 and the others as 0, running one model for each posture via gamm command [57]. We considered the instantaneous sample points of play for which we recorded the posture (n = 951) as a statistical unit (i.e., for each instantaneous sample point of play where we noted the focal animal’s posture), as individuals can change the posture during the same play event. For age, we used full restricted maximum likelihood method for model selection, tensor product smooth and penalized regression spline [58].

We used a Generalized Linear Mixed Model (GLMM) to assess whether the frequency of play events was influenced by the environmental conditions (i.e., occurrence of rain and moonlight), the age of the focal individual (days), sex and the group size (number of possible play partners). We used the day of observation (n = 1136) as a statistical unit (i.e., number of instantaneous sample points of play per day of observation) and considered the days when we did not observe the individuals play. To control for observation effort, we included the duration of observations in the model, and, to account for individual variation, we included individual as a mixed factor. We used the function gamlss for running the GLMM, and the function get MoonIllumination to record the percentage of the lunar disc illuminated. We used the zero-adjusted Inverse Gaussian distribution with link function log. We selected the best fit model using the Akaike Information Criteria (AIC) [59]. We considered the best fit as the model with the lowest AIC. For the statistical analysis, we used the R-packages mgcv for the GAMM, gamlss for the GLMM, suncalc for obtaining the moonlight data, and ggplot2 for plotting the graphs [60,61]. We considered *p* = 0.05 as significance level and used R version 3.5.1 [57].

## Figures and Tables

**Table 1:** Results of the Generalized Additive Mixed Models of the factors (relationship between play partners and age) influencing the posture of play behavior in Javan slow loris (*Nycticebus javanicus*). Family of distribution: binomial. Data are based on 951 instantaneous data points collected between April 2012 and December 2020 during which the posture during play was collected. The reference category for the relationship between play partners is sibling. The category other refers to play between pair mates or between offspring and both parents.

Response Variable	Predictor	Estimate	Std. Error	*t*-Value	*p*	Smooth Term		*p*
						Edf	F-Value	
Horizontal suspension	Intercept	1.467	0.219	6.7	<0.001 *			
	Adult male—offspring	−0.177	0.254	−2.5	0.485			
	Mother—offspring	−0.785	0.261	−3.0	0.003 *			
	Other	−0.923	0.319	−2.9	0.004*			
	(Age)					1.000	11.4	<0.001 *
Non-suspensory	Intercept	−2.041	0.271	−7.5	<0.001 *			
	Adult male—offspring	−0.245	0.331	−0.7	0.460			
	Mother—offspring	0.479	0.328	1.5	0.144			
	Other	1.407	0.368	3.8	<0.001 *			
	(Age)					1.748	13.3	<0.001 *
Vertical suspension	Intercept	−2.897	0.347	−8.3	<0.001 *			
	Adult male ‒offspring	0.608	0.368	1.7	0.099			
	Mother—offspring	1.021	0.382	2.7	0.008 *			
	Other	−1.954	1.080	−1.8	0.071			
	(Age)					1.943	2.5	0.081

* *p* < 0.05.

**Table 2:** Results of the Generalized Linear Mixed Model of the biological and environmental factors influencing the frequency of play of Javan slow loris (*Nycticebus javanicus*). ΔAIC = 42.11, i.e., the difference between the AIC of the first ranked model and the AIC of the null model. Data are based on 1137 days of data collection (considering also days when the individuals did not play) between April 2012 and December 2020.

Response Variable ^a^	Predictor Variables	Estimate	Std. Error	*t*-Value	*p*
Frequency of play	Intercept	1.091	0.109	10.1	<0.001 *
	N observations (effort control)	0.005	0.002	−2.3	0.03 *
	Rain	0.026	0.006	4.2	<0.001 *
	Moonlight	0.012	0.003	3.9	<0.001 *
	Age	−0.0002	0.00001	96.6	<0.001 *

^a^ Zero-adjusted inverse Gaussian distribution (link function log). * *p* < 0.05.

## Data Availability

The data presented in this study are available on request from the corresponding author. The data are not publicly available due to restrictions in relation to the permits issued to work in Indonesia.

## Appendix A

**Table A1:** Observation time (i.e., hours of direct contact with animals), number of instantaneous sample points of play (every 5 min) recorded, sex, and age range of the 56 individuals followed at Cipaganti, West Java, Indonesia, between 2012 and 2020.

ID	Sex	Age (Range in Days)	Observation Time (h)	Instantaneous Sample Points of Play
acil	M	1708–2593	143	17
alomah	M	731–1589	209	52
azka	M	1934–3655	109	15
charlie	F	1963–2529	31	8
dali	M	375–483	10	1
dempak	M	905–1039	33	1
dindi	M	784–1393	223	0
ena	F	2160–3644	98	9
endor	F	186	1	5
fernando	M	1280–3578	448	65
galaksi	F	131–457	7	7
ghee	F	192–297	29	5
guntur	M	904–1139	39	13
indomi	F	453–538	48	0
jaimi	M	406–896	226	2
jogja	M	1934–2004	10	2
lalit	M	133–558	193	131
lava	F	108–210	2	2
ln	M	211–770	261	39
loopi	F	172–981	115	36
lucu	F	193–3139	572	80
lupak	F	79–1585	316	99
maaf	M	361–541	34	17
maya	F	530–1411	110	26
mikio	M	67–1128	46	19
mimi	F	839–1535	250	2
mo	M	724–1277	34	2
mungkin	M	126–787	112	34
oneeye	F	1999–4013	154	14
oniks	M	274	1	1
opal	F	100–118	3	8
pakbulan	M	1151	2	1
rufio	M	654–1993	355	99
sempurna	F	290–897	38	5
shakti	F	223–616	109	78
shanti	M	286–542	120	50
shirley	F	1930–4707	419	17
sibau	F	812–2285	63	8
sirious	M	71	1	7
smol	F	352–514	84	20
solo	M	173–726	94	52
sri	M	258	1	1
stan	M	130	1	1
star	F	348–535	139	76
tahini	F	170–596	5	12
tereh	F	1461–3609	236	28
timone	F	292	2	1
tombol	F	209–943	215	70
toyib	M	927–2399	72	6
tyrion	M	188	2	1
tzatziki	M	161–538	53	3
utari	M	196–317	3	2
xandie	M	164–320	34	8
xena	F	1282–2077	84	15
xerxes	M	569–664	38	0
yogi	M	320–575	7	8
**TOTAL**	**25 F, 31 M**	**67–4707**	**6040**	**1283**
